# Mice lacking the cAMP effector protein POPDC1 show enhanced hippocampal synaptic plasticity

**DOI:** 10.1093/cercor/bhab426

**Published:** 2021-12-23

**Authors:** Mahesh Shivarama Shetty, Laurence Ris, Roland F R Schindler, Keiko Mizuno, Laura Fedele, Karl Peter Giese, Thomas Brand, Ted Abel

**Affiliations:** Department of Neuroscience and Pharmacology, Carver College of Medicine, University of Iowa, Iowa City, IA 52242, USA; Iowa Neuroscience Institute, Carver College of Medicine, University of Iowa, Iowa City, IA 52242, USA; Department of Neuroscience, University of Mons, Research Institute for Health Sciences and Technology, 7000 Mons, Belgium; National Heart and Lung Institute, Imperial College London, London W12 ONN, UK; Department of Neuroscience, King’s College, London SE5 9NU, UK; National Heart and Lung Institute, Imperial College London, London W12 ONN, UK; Department of Neuroscience, King’s College, London SE5 9NU, UK; National Heart and Lung Institute, Imperial College London, London W12 ONN, UK; Department of Neuroscience and Pharmacology, Carver College of Medicine, University of Iowa, Iowa City, IA 52242, USA; Iowa Neuroscience Institute, Carver College of Medicine, University of Iowa, Iowa City, IA 52242, USA

**Keywords:** BVES, cAMP signaling, LTP, memory, phosphodiesterases

## Abstract

Extensive research has uncovered diverse forms of synaptic plasticity and an array of molecular signaling mechanisms that act as positive or negative regulators. Specifically, cyclic 3′,5′-cyclic adenosine monophosphate (cAMP)-dependent signaling pathways are crucially implicated in long-lasting synaptic plasticity. In this study, we examine the role of Popeye domain-containing protein 1 (POPDC1) (or blood vessel epicardial substance (BVES)), a cAMP effector protein, in modulating hippocampal synaptic plasticity. Unlike other cAMP effectors, such as protein kinase A (PKA) and exchange factor directly activated by cAMP, POPDC1 is membrane-bound and the sequence of the cAMP-binding cassette differs from canonical cAMP-binding domains, suggesting that POPDC1 may have an unique role in cAMP-mediated signaling. Our results show that *Popdc1* is widely expressed in various brain regions including the hippocampus. Acute hippocampal slices from *Popdc1* knockout (KO) mice exhibit PKA-dependent enhancement in CA1 long-term potentiation (LTP) in response to weaker stimulation paradigms, which in slices from wild-type mice induce only transient LTP. Loss of POPDC1, while not affecting basal transmission or input-specificity of LTP, results in altered response during high-frequency stimulation. *Popdc1* KO mice also show enhanced forskolin-induced potentiation. Overall, these findings reveal POPDC1 as a novel negative regulator of hippocampal synaptic plasticity and, together with recent evidence for its interaction with phosphodiesterases (PDEs), suggest that POPDC1 is involved in modulating activity-dependent local cAMP–PKA–PDE signaling.

## Introduction

Understanding the molecular mechanisms involved in learning and memory represents a significant challenge in neuroscience. Studies over the years have identified activity-dependent synaptic plasticity as one of the key cellular mechanism of memory (Neves et al. 2008). Extensive research, both in invertebrate and vertebrate species has uncovered diverse forms of synaptic plasticity and the underlying complex molecular signaling pathways. Interestingly, this has revealed the existence of both positive and negative regulators of synaptic plasticity and memory (Abel et al. 1998).

The cyclic 3′,5′-cyclic adenosine monophosphate (cAMP)-dependent signaling pathway plays a crucial role in persistent forms of synaptic plasticity (Frey et al. 1993; Abel et al. 1997). cAMP signaling is involved in a wide variety of cellular processes and subject to extensive spatiotemporal control to achieve signal- and cell type-specific responses. Subcellular compartment-specific nanodomain signaling complexes are formed in cells with the help of multiple A-kinase anchor proteins (AKAPs) (Bauman et al. 2004), which bind cAMP effector proteins such as protein kinase A (PKA) or exchange factor directly activated by cAMP (EPAC), adenylyl cyclases, phosphodiesterases (PDEs), and target proteins (Houslay 2010; Schleicher and Zaccolo 2020; Zaccolo et al. 2021). The concerted actions of these proteins lead to the transient activation of downstream molecular targets such as the α-amino-3-hydroxy-5-methyl-4-isoxazolepropionic acid (AMPA) receptor or the voltage-gated calcium channel Ca_V_1.2 in response to specific patterns of synaptic activity (Patriarchi et al. 2018). The cAMP signaling pathway also induces activity-dependent gene expression required for the persistence of synaptic plasticity and memory (Nguyen et al. 1994; Deisseroth et al. 1996; Impey et al. 1996; Abel and Nguyen 2008; Kandel et al. 2014).

The Popeye domain-containing (POPDC) proteins are a novel class of cAMP effector proteins characterized by an evolutionarily conserved Popeye domain (PF04831) that functions as a high-affinity cAMP-binding domain (Schindler and Brand 2016; Brand 2018; Swan et al. 2019). The family comprises three members: POPDC1 (also known as blood vessel epicardial substance (BVES)), POPDC2, and POPDC3 (Andree et al. 2000). POPDC1 is the most studied protein among the isoforms. The POPDC proteins display overlapping but isoform-specific expression patterns (Froese et al. 2012). Although POPDC genes are abundantly expressed in heart and skeletal muscle, where they have been primarily studied, they are also found in the central and autonomic nervous system (Andree et al*.* 2000; Vasavada et al. 2004; Hager and Bader 2009). POPDC proteins have a short extracellular domain followed by three transmembrane domains (Schindler and Brand 2016; Brand 2018; Swan et al*.* 2019). The carboxy-terminal domain of POPDC proteins is isoform-specific, variable in length, contains regions of low complexity and is predicted to be partly disordered (Brand 2019). Constitutive knockouts (KO) of *Popdc1* and *Popdc2* have been engineered in mice, which show cardiac arrhythmia (stress-induced sinus node bradycardia) and impaired muscle regeneration (Andrée et al. 2002; Froese et al*.* 2012). Likewise, patients carrying mutations in *POPDC1*, *POPDC2*, or *POPDC3* develop cardiac arrhythmia (AV-block) and/or muscular dystrophy phenotypes (Schindler et al. 2016; De Ridder et al. 2019; Vissing et al. 2019; Indrawati et al. 2020; Rinne et al. 2020).

Although expression of POPDC1 in the brain has been reported previously (Andree et al. 2000; Vasavada et al. 2004; Hager and Bader 2009), until now, to our knowledge no study has specifically looked into the expression pattern or determined the function of POPDC1 in the nervous system. Given the diverse roles of the cAMP signaling in the nervous system, we predict that POPDC1 might have specific functions in the modulation of cAMP signaling events in neurons. The fact that the phosphate-binding cassette of the Popeye domain differs from canonical ones further suggests a unique role, which differs from that of other cAMP effectors. Additionally, some of the POPDC1-interacting proteins identified thus far, such as the two-pore potassium channel TREK-1 (Froese et al. 2012), the synaptobrevins VAMP2 and 3 (Hager et al. 2010), zonula occludens-1 (ZO1) (Osler et al. 2005), dystrophin (Schindler et al*.* 2016), and N-myc downstream-regulated gene 4 (NDRG4) (Benesh et al. 2013), have all been implicated in neuronal function. This suggests that in addition to its role in striated muscle, POPDC1 might also have specific functions in neuronal tissue. Here, we investigated the role of POPDC1 in hippocampal synaptic plasticity using constitutive *Popdc1* KO mice. Our results show that *Popdc1* is prominently expressed in the hippocampus among other brain regions, and *Popdc1* KO mice display enhancements in specific forms of hippocampal synaptic plasticity. These findings provide the first evidence for an important role of the cAMP effector protein POPDC1 in hippocampal function.

## Materials and Methods

All animal procedures were carried out in accordance with National Institutes of Health regulations for the care and use of animals in research and were approved by the Institutional Animal Care and Use Committees at the University of Iowa, Imperial College London, University of Mons, and King’s College London.

### Popdc1 Knockout Mice

The generation of the mouse *Popdc1* KO mutant has been described previously (Andrée et al*.* 2002). Briefly the coding sequence of *Popdc1* was substituted by a *lacZ* reporter gene carrying a nuclear localization signal (NLS), through homologous recombination. The *Popdc1* KO mutation is kept on a C57BL/6 J background.

### X-gal Staining

Brains were harvested from 3-months old male heterozygous *Popdc1-LacZ* mutant mice and fixed with 2% (v/v) formaldehyde, 0.2% (v/v) glutaraldehyde, 0.02% (v/v) Nonidet P-40 (NP-40), 0.01% (v/v) sodium deoxycholate in phosphate-buffered saline (PBS) for 4 h at 4 °C. The fixed brains were washed three times with PBS and then incubated at 4 °C with 20% and 30% sucrose. The brains were mounted on a cryomold with optimal cutting temperature (OCT) compound and stored at −80 °C until sectioning. Cryosections of 20–40 μm thickness were mounted on glass slides (Superfrost plus). Sections were incubated with staining buffer (2 mM MgCl_2_, 0.01% (v/v) sodium deoxycholate, 0.02% NP40) and then stained with 5-bromo-4-chloro-3-indolyl-b-D-galactopyranoside (X-Gal) at 1 mg/mL in staining buffer containing 5 mM K_3_[Fe(CN)_6_] and 5 mM K_4_[Fe(CN)_6_] at 37 °C until color has developed. Sections were counterstained with 0.1% Nuclear Fast Red in 5% aluminum sulfate, dehydrated through an ascending series of ethanol, immersed in xylene, and coverslipped with Entellan (E. Merck, Switzerland).

### Synaptosomal Purification and Western Blot Analysis

All steps of crude synaptosomal preparation were performed as described (Tiwari et al. 2016). Briefly, frozen hippocampi from an adult male wild-type (WT) mouse were homogenized (10 strokes, 750 rpm) in 20 μL/mg homogenization buffer (0.32 M sucrose, 1 mM NaHCO_3_, 1 mM MgCl_2_, 10 mM HEPES, pH 7.4). Following centrifugation at 381 × *g* for 10 min at 4 °C to remove nuclei and cell debris, the resulting supernatant was centrifuged at 16 089 × *g* for 15 min to obtain a crude synaptosomal pellet. Synaptosomal pellets (P2 fraction) were resuspended in 300 μL homogenization buffer for each 50 mg of starting tissue. The supernatant (S2 fraction) contained the cytoplasmic proteins. Protein concentration of each sample was determined using Bradford reagent. Proteins were size separated by gel electrophoresis and transferred onto nitrocellulose membrane (BioTrace NT, Pall). The membrane was washed with TBST (50 mM Tris-HCl, pH 7.4, 150 mM NaCl, 0.1% [v/v] Tween-20) and blocked in 5% (w/v) low-fat milk in TBST for 1 h at room temperature and subsequently incubated with POPDC1 antibody (sc-49 889, Santa Cruz Biotechnology Inc.) overnight at 4 °C. After several washes, the blots were incubated for 1 h at room temperature with horseradish peroxidase-coupled anti-goat antibody (PI-9500, Vector Laboratories). After washing, signals were detected using a Chemidoc gel imaging system (BioRad).

### Electrophysiology

Homozygous *Popdc1* mutants and littermate WT mice (both male and female) of 2–5 months of age were used. Mice were euthanized by cervical dislocation and the brain was quickly dissected into cold artificial cerebrospinal fluid (aCSF) (124 mM NaCl, 4.4 mM KCl, 1 mM NaH_2_PO_4_, 2.5 mM CaCl_2_.2H_2_O, 1.3 mM MgSO_4_.7H_2_O, 26.2 mM NaHCO_3_, and 10 mM D-glucose; pH ~7.4) being continuously bubbled with carbogen (95% O_2_, 5% CO_2_). Isolation of hippocampi and preparation of acute hippocampal slices was performed as described (Shetty et al. 2015). Transverse acute hippocampal slices of 400 μm thickness were prepared from both hippocampi using a manual McIlwain slicer (Stoelting, Wooddale, IL, USA). The slices were quickly transferred onto a net insert in an interface recording chamber (Fine Science Tools, Foster City, CA) and incubated at 28 °C in a humidified carbogen atmosphere for at least 2–3 h before starting the recordings. The slices were perfused at 1 mL/min with oxygenated aCSF throughout the experiments. Field excitatory postsynaptic potentials (fEPSPs) were recorded in CA1 stratum radiatum by stimulating Schaffer collaterals with a monopolar, lacquer coated stainless-steel electrode (#571000, A-M Systems) and recording with an aCSF-filled glass microelectrode (2–5 MΩ resistance). Test stimulation was a biphasic, constant current pulse (100 μs duration per phase) delivered every minute at a stimulation intensity that evoked ~40% (or ~50% in long-term depression [LTD] experiments) of the maximal fEPSP amplitude as determined by an input–output curve (stimulation intensity vs. fEPSP amplitude) in each experiment. In all experiments, a stable baseline was recorded for at least 20 min before LTP induction or drug application. Paired-pulse facilitation (PPF) was examined at various interpulse intervals (300–25 ms).

### Synaptic Plasticity Paradigms

The 1-train LTP stimulation comprised of a single 100 Hz train (1 s duration; pulse width 0.1 ms per phase). Spaced 4-train LTP paradigm comprised of four 100 Hz, 1 s trains (pulse width 0.1 ms per phase) delivered with an intertrain interval of 5 min. Weak theta-burst LTP stimulation (TBS) consisted of two bursts delivered at 5 Hz with each burst containing four pulses delivered at 100 Hz (pulse width 0.1 ms per phase). For LTD experiments, 2–3 months old mice (both male and female) were used. LTD was induced by 1 Hz stimulation for 15 min (900 pulses; pulse width 0.1 ms per phase). All stimulation protocols were delivered at the baseline stimulation intensity. Forskolin (FSK; Sigma-Aldrich; #F3917) was prepared as a 50 mM stock in dimethyl sulfoxide (DMSO) and bath applied at a final concentration of 50 μM in aCSF for 15 min (final DMSO concentration of 0.1%). The phosphodiesterase inhibitor 3-Isobutyl-1-methylxanthine (IBMX) (Sigma-Aldrich; #I5879) was prepared as a 90 mM stock in DMSO and bath applied at a final concentration of 30 μM in aCSF for 15 min. The PKA inhibitor KT5720 (Tocris; #1288) was prepared as a 1 mM stock in dimethyl sulfoxide (DMSO) and bath applied at a final concentration of 1 μM in aCSF for 30 min (final DMSO concentration of 0.1%). As a vehicle control, 0.1% DMSO in aCSF was used.

### Electrophysiology Data Analysis

Data were acquired using Clampex 10 and Axon Digidata 1440/1550 digitizer (Molecular Devices, Union City, CA) at 20 kHz and were low-pass filtered at 2 kHz with a four-pole Bessel filter. Data analysis was performed using Clampfit 10. Data plots and statistical analysis was performed using GraphPad Prism 9. In all the electrophysiological data, “*n”* represents the number of mice, except in PKA inhibitor experiments where “*n*” represents the number of slices (comparison within the same genotype). For each slice, the fEPSP slopes were normalized against the average slope over the 20 min baseline. Data from replicate slices from the same mouse were averaged when comparing between genotypes. Data distribution was checked by normality tests. Induction of LTP was assessed at earlier durations immediately or shortly after LTP-inducing stimuli. The maintenance of LTP was compared between two groups using independent *t*-tests on the last 20-min of the recordings. Input–output and PPF data were analyzed using two-way repeated-measures ANOVA with Šídák’s test for multiple comparisons. Data are presented as mean ± standard error mean (SEM). Differences were considered statistically significant when alpha (*P*) ≤ 0.05.

*1-train tetanus trace analysis*: The fEPSP responses during the 1-train stimulation (100 Hz, 1 s; 100 stimuli) were analyzed to assess the fEPSP suppression dynamics. The amplitude of the responses to first 15 stimuli were plotted as percentage changes from the first response and compared using two-way repeated measures ANOVA. Tetanus train data were reduced by decimation to 1 ms sampling interval on Clampfit 10 to get rid of the stimulation artifacts. The area under the curve, maximum rise slope, and decay slopes were calculated for the entire train response after data reduction. Data from multiple slices of the same mouse were averaged. Data are presented as mean ± SEM.

### cAMP Enzyme-Linked Immunosorbent Assay (ELISA) Assay

*Popdc1* KO and WT littermate mice of 2–3 months age (both male and female) were euthanized by cervical dislocation and the hippocampal slices were prepared as described for the electrophysiology experiments. Slices from each mouse were incubated in two independently perfusable wells of the same interface incubation chamber (same chambers used for the electrophysiology experiments). Following 2 h of recovery incubation in oxygenated aCSF, FSK (50 μM) or vehicle (0.1% DMSO) dissolved in aCSF was bath applied to the slices for 15 min. Ten minutes after the end of the drug application, slices were quickly collected in a prechilled Eppendorf tube on dry ice, flash frozen in liquid nitrogen and stored at −80 °C. On the day of the assay, samples were homogenized in 0.1 M HCl using stainless steel beads. The homogenate was centrifuged at 600 × *g* for 10 min and the supernatant was immediately processed in triplicates for cAMP ELISA using the direct cAMP ELISA kit for intracellular cAMP quantification (#ADI-900-066; Enzo Life Sciences, Farmingdale, NY, USA) following manufacturer’s instructions. The cAMP levels were normalized to the total protein concentration in the supernatant determined by Bradford Assay using BSA standard curve (Bio-Rad). The final cAMP concentration is expressed as pmol cAMP per mg of total protein. The vehicle-treated slices are denoted as “Basal” group and the FSK-treated slices are denoted as “FSK” group. The differences between the basal and FSK groups in WT and *Popdc1* KO mice were analyzed by two-way ANOVA followed by Šídák’s multiple comparisons tests.

## Results

### POPDC1 is Expressed in the Hippocampus and is Enriched in Crude Synaptosomes

**Figure 1:**
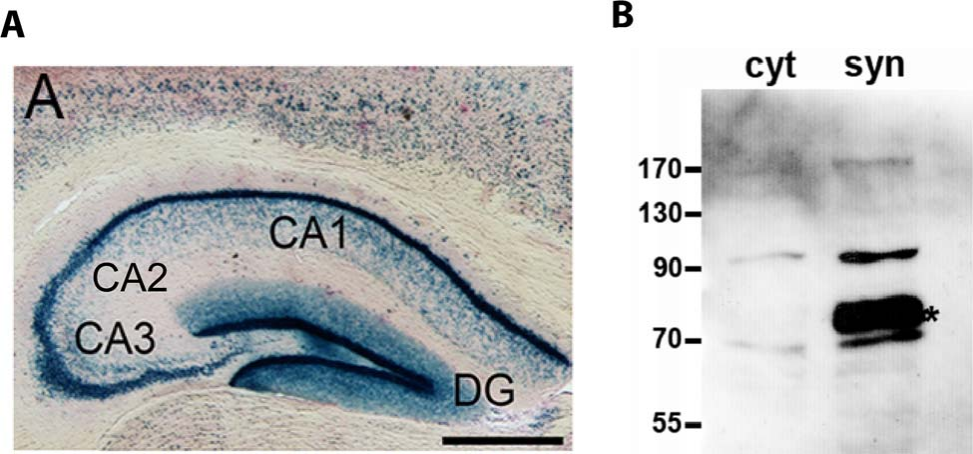
*Popdc1* is expressed in all the hippocampal subregions and the protein seems to be enriched near the synapses. (*A*) β-galactosidase staining of a coronal brain section of an adult male heterozygous *Popdc1*-*LacZ* mouse. Only the hippocampal region is depicted. (*B*) Western blot analysis of cytoplasmic fraction (cyt) and crude synaptosomes (syn) isolated from the hippocampi of an adult male WT mouse. Asterisk demarcates the immunoreactive band for POPDC1. CA1, CA2, CA3: Cornu Ammonis areas 1, 2, and 3; DG: dentate gyrus. Scale bar in (*A*): 0.5 mm.

The expression of *Popdc1* in the hippocampus was investigated by staining for beta galactosidase using coronal brain sections of adult male heterozygous *Popdc1*-*LacZ* mutant mice, which carry a NLS-*LacZ*-reporter allele in the *Popdc1* locus (Andrée et al. 2002). A strong expression of *Popdc1* in the hippocampal CA1, CA2, CA3 and dentate gyrus (DG) regions was observed (Fig. 1*A*). Expression of *Popdc1* was also seen in other brain regions including cerebral cortex, piriform area, entorhinal cortex, amygdala, olfactory tubercle, caudoputamen, and in several nuclei in the midbrain and medulla (see Supplementary Fig. S1). We then performed a preliminary assessment of the subcellular localization of POPDC1 in the hippocampus of a WT mouse and found that it was preferentially enriched in the crude synaptosomal fraction. A prominent immunoreactive band at approximately 75 kDa was present in the synaptosomal fraction but not in the cytosolic fraction (Fig. 1*B*). The higher molecular weight of the immunoreactive band in brain tissue compared with the heart is probably due to tissue-specific glycosylation as previously suggested (Vasavada et al*.* 2004). This suggests that POPDC1 might be enriched in the membranes near the synapses but evidence from additional approaches such as immunohistochemistry is warranted.

### Popdc1 KO Mice Show PKA-Dependent Enhancement in LTP in Response to Submaximal High-Frequency Stimulation

**Figure 2:**
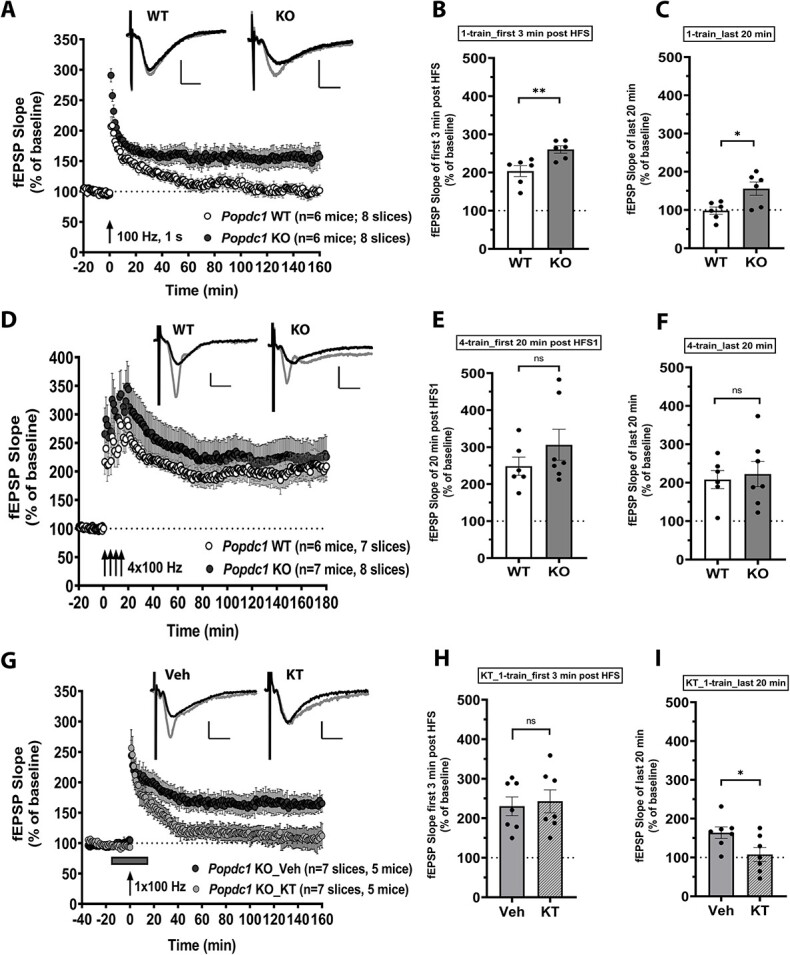
*Popdc1* KO mice show PKA-dependent long-lasting LTP in response to submaximal high-frequency stimulation. (*A*) LTP induced by a single 100 Hz train (1-train LTP) is persistently enhanced in slices from *Popdc1* KO mice (*n* = 6 mice; 4 males, 2 females) compared with WT littermates (*n* = 6 mice; 2 males, 4 females). (*B*) Induction of LTP assessed by the mean fEPSP slope over first 3 min after HFS is significantly higher in *Popdc1* KO compared with WT; unpaired *t*-test, two-tailed, *t* = 3.281, df = 10, *P* = 0.008. (*C*) The mean fEPSP slope over the last 20 min recordings is significantly higher in *Popdc1* KO compared with WT; unpaired *t*-test, two-tailed, *t* = 2.897, df = 10, *P* = 0.0159. (*D*) Persistent LTP induced by repeated 100 Hz stimulation trains (spaced-4-train protocol) is similar in slices from *Popdc1* KO mice (*n* = 7 mice; 4 males, 3 females) compared with WT littermates (*n* = 6 mice; 4 males, 2 females). (*E*) Induction of LTP assessed by the mean fEPSP slope over first 20 min after HFS1 is slightly higher in *Popdc1* KO compared with WT; unpaired *t*-test, two-tailed, *t* = 1.141, df = 11, *P* = 0.278. (*F*) The mean fEPSP slope over the last 20 min recordings is similar in the *Popdc1* KO compared with WT; unpaired *t*-test, two-tailed, *t* = 0.3464, df = 11, *P* = 0.7356. (*G*) The enhanced 1-train LTP in slices from *Popdc1* KO mice is dependent on PKA activation. Bath application of the PKA inhibitor KT5720 (1 μM) during LTP induction prevents the LTP enhancement (*n* = 7 slices; 5 mice; 4 males, 1 female), whereas DMSO vehicle (Veh) treatment does not (*n* = 7 slices; 5 mice; 2 males, 3 females). (*H*) LTP induction assessed by the mean fEPSP slope over first 3 min after HFS is similar in vehicle or KT-treated slices; unpaired *t*-test, two-tailed, *t* = 0.3347, df = 12, *P* = 0.744. (*I*) The mean fEPSP slope over the last 20 min recordings is significantly lower after KT5720 treatment compared with slices receiving vehicle; unpaired *t*-test, two-tailed, *t* = 2.396, df = 12, *P* = 0.0338. In A, D, and G, representative fEPSP traces during the baseline (black traces) and at the end of the recording period (gray traces) for each group are shown. Calibration bars for all the traces: 2 mV vertical; 5 ms horizontal. Errors bars represent SEM.

We next examined the impact of the loss of POPDC1 on long-term potentiation (LTP) at the hippocampal CA1 Schaffer collateral synapses using acute slices. The Schaffer collateral fibers were stimulated and the field-EPSP responses were recorded in the CA1 stratum radiatum. In slices from WT animals, stimulation with a single train of 100 Hz, 1 s protocol elicited a transient potentiation (1-train LTP) (Fig. 2*A*). It is thought that 1-train LTP is independent of both protein synthesis and PKA activation, and decays to baseline levels within 1–2 h (Frey et al. 1993; Huang and Kandel 1994; Nguyen and Woo 2003). Interestingly, the same protocol resulted in enhanced LTP in slices from *Popdc1* KO mice (Fig. 2*A*). The potentiation was significantly higher (two-tailed unpaired *t*-test, *P* = 0.008) immediately following induction (Fig. 2*B*; average of first 3 min after high-frequency stimulation (HFS)) in the KO slices (260.3 ± 9.6%) compared with the WT slices (203.8 ± 14.3%). Although 10–15 min after induction there was no significant difference between the two groups (WT: 152.8 ± 7.7%; KO: 176.6 ± 8.7%, two-tailed unpaired *t*-test, *P* = 0.067), the potentiation persisted in the KO slices while it decayed to baseline levels in the WT slices. Maintenance of LTP assessed over the last 20 min of recording showed significantly enhanced potentiation in the KO slices (155.7 ± 17.3%) compared with WT slices (98.3 ± 9.6%) (Fig. 2*C*). In an additional set of experiments, we confirmed that this LTP enhancement was input-specific because a control pathway recorded in the same slices showed no nonspecific potentiation (see Supplementary Fig. S2) and hence suggesting that loss of POPDC1 did not result in altered input specificity of LTP. Next, we assessed LTP induced by a stronger protocol comprising four 100 Hz, 1 s trains spaced at 5 min (spaced 4-train LTP). This stimulation paradigm induces a saturating LTP that is long-lasting and is dependent on cAMP–PKA signaling, transcription, and translation (Huang and Kandel 1994; Nguyen et al*.* 1994; Abel et al*.* 1997; Malleret et al. 2001). The spaced 4-train LTP was similar and nondecremental in slices from both the *Popdc1* KO and WT littermates (Fig. 2*D*). Although the potentiation over the course of induction (20 min from the first train) was slightly higher in the KO slices (306.3 ± 41.8%) compared with the WT slices (248.3 ± 24.7%), the increase was not statistically significant (*t*-test, *P* = 0.278; Fig. 2*E*). Similar trend was also observed for potentiation shortly after the induction (35–40 min after first train; *t*-test, *P* = 0.299). Maintenance of LTP assessed over the last 20 min of recording showed similar persistent potentiation in both KO (222.6 ± 32.8%) and WT slices (208.1 ± 23.6%) (Fig. 2*F*). These results show that *Popdc1* KO mice exhibit enhanced LTP in response to submaximal high-frequency stimulation.

Because PKA activation is crucially involved in long-lasting forms of LTP (Abel et al. 1997; Nguyen and Woo 2003), we then investigated the PKA dependence of the persistent 1-train LTP seen in the *Popdc1* KO mice. We bath applied a cell-permeable competitive PKA inhibitor, KT5720 (Kase et al. 1987) to the *Popdc1* KO slices at a concentration of 1 μM during the 1-train LTP stimulation (15 min before and 15 min after) and found that it blocked the enhancement in LTP compared with the DMSO vehicle-treated KO slices (Fig. 2*G*). At the concentration used, KT5720 (KT) has been shown not to affect basal field-EPSP responses (Chen et al. 2003; Lu et al. 2007). The induction of LTP (over the first 3 min after the train; Fig. 2*H*) was not different between the KT-treated slices (242.9 ± 29%) and vehicle-treated slices (230.4 ± 23.5%). Shortly after the induction (10–15 min after the train), potentiation in the KT-treated slices showed a trend for decay (172.9 ± 16.9%) compared with the vehicle-treated slices (204.5 ± 19.1%). Towards the end of the recording period (last 20 min), KT-treatment completely blocked the enhanced LTP in the KO slices (107.9 ± 18.1%) compared with the vehicle-treatment (163.9 ± 14.9%) (Fig. 2*I*). These results suggest that the enhanced LTP seen in the *Popdc1* KO mice requires PKA activity.

### Basal Synaptic Transmission and Short-Term Plasticity are not Altered but Response During High-Frequency Stimulation is Altered in Popdc1 KO Mice

**Figure 3:**
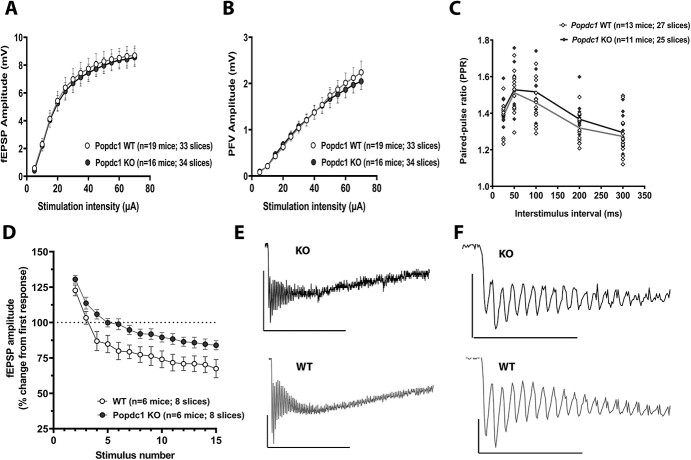
*Popdc1* KO mice show no alterations in basal synaptic transmission and PPF but show an altered response during high-frequency stimulation. (*A*) Input–output relation plot of the fEPSP amplitude versus the incremental stimulation intensity in *Popdc1* KO mice (*n* = 16 mice; 6 females, 10 males) and WT littermates (*n* = 19 mice; 9 females; 10 males). Two-way repeated measures ANOVA showed no significant differences between the genotypes F (1,33) = 0.064; *P* = 0.801. (*B*) Input–output relation plot of the PFV amplitude versus the incremental stimulation intensity in *Popdc1* KO mice (*n* = 16 mice; 6 females, 10 males) and WT littermates (*n* = 19 mice; 9 females; 10 males). Two-way repeated measures ANOVA showed no significant differences between the genotypes *F* (1,33) = 0.044; *P* = 0.835. (*C*) PPF (calculated as PPR = amplitude of fEPSP2/amplitude of fEPSP1) measured over a range of interpulse intervals (25–300 ms) in *Popdc1* KO mice (*n* = 11 mice; 5 females, 6 males) and WT littermates (*n* = 13 mice; 7 females; 6 males). Two-way repeated measures ANOVA showed no significant differences between the genotypes *F* (1,22) = 1.061; *P* = 0.3141. (*D*) Field-EPSP response to first 15 stimuli of the 1-train HFS (100 Hz, 1 s). The amplitude of each response is plotted as the percentage change from the first response. The progressive fEPSP suppression seen in WT slices is significantly dampened in *Popdc1* KO slices (two-way RM-ANOVA, genotype effect: *F* (1,11) = 6.601, *P* = 0.026). (*E*) Representative fEPSP response traces during the entire 1-train stimulation in WT and KO mice. Calibration: 1 mV, 500 ms. (*F*) The traces in (*E*) on an expanded scale to highlight the first 15 responses. Calibration: 1 mV, 100 ms. Errors bars represent SEM.

Next, we examined whether the loss of *Popdc1* alters basal synaptic transmission in hippocampal neurons by comparing input–output responses. The stimulus–response curves evoked with progressively increasing stimulus intensities (5–70 μA) showed no significant differences both in the field-EPSP amplitudes (Fig. 3*A*) and in presynaptic fiber volley (PFV) amplitudes (Fig. 3*B*) between the WT and *Popdc1* KO mice. We also investigated PPF, a form of short-term synaptic plasticity and an index of presynaptic activity and release probability where two stimuli given in close succession lead to the facilitation in the response to the second stimuli (Zucker and Regehr 2002; Jackman and Regehr 2017). At all the interstimulus intervals tested (ranging from 300 to 25 ms), no significant differences in the facilitation, calculated as paired-pulse ratio (PPR = Amplitude of fEPSP2/Amplitude of fEPSP1), was observed between slices from WT and *Popdc1* KO animals (Fig. 3*C*). To further investigate the impact of the loss of POPDC1 on neuronal excitability, we analyzed the fEPSP responses during the high-frequency tetanic stimulation for 1-train LTP induction. Analysis of the fEPSP amplitudes in response to the first 15 stimuli in the train revealed a significantly dampened fEPSP suppression dynamics during the HFS train in the *Popdc1* KO slices compared with the WT slices (Fig. 3*D*; two-way RM-ANOVA, genotype effect, *P* = 0.026). Analysis of the entire cumulative train response (Fig. 3*E*) showed that averaged maximum rise slope was significantly smaller in the *Popdc1* KO slices compared with the WT slices (see Supplementary Fig. S3*A*; *t*-test, *P* = 0.0496), whereas the maximum decay slope was similar in both (see Supplementary Fig. S3*B*; *t*-test, *P* = 0.938). The average cumulative area under the curve for the entire train duration was slightly smaller in the *Popdc1* KO slices compared with the WT slices, but the difference was not statistically significant (see Supplementary Fig. S3*C*; *t*-test, *P* = 0.295).

Together, these results indicate that POPDC1 seems to have a role in neuronal excitability and the impact of the loss of POPDC1, while not apparent during responses to low-rate basal synaptic stimulation, becomes evident during response to high-frequency stimuli.

### Popdc1 KO Mice Show Enhancement in Potentiation Induced by Forskolin but not with Simultaneous Inhibition of Phosphodiesterases

**Figure 4:**
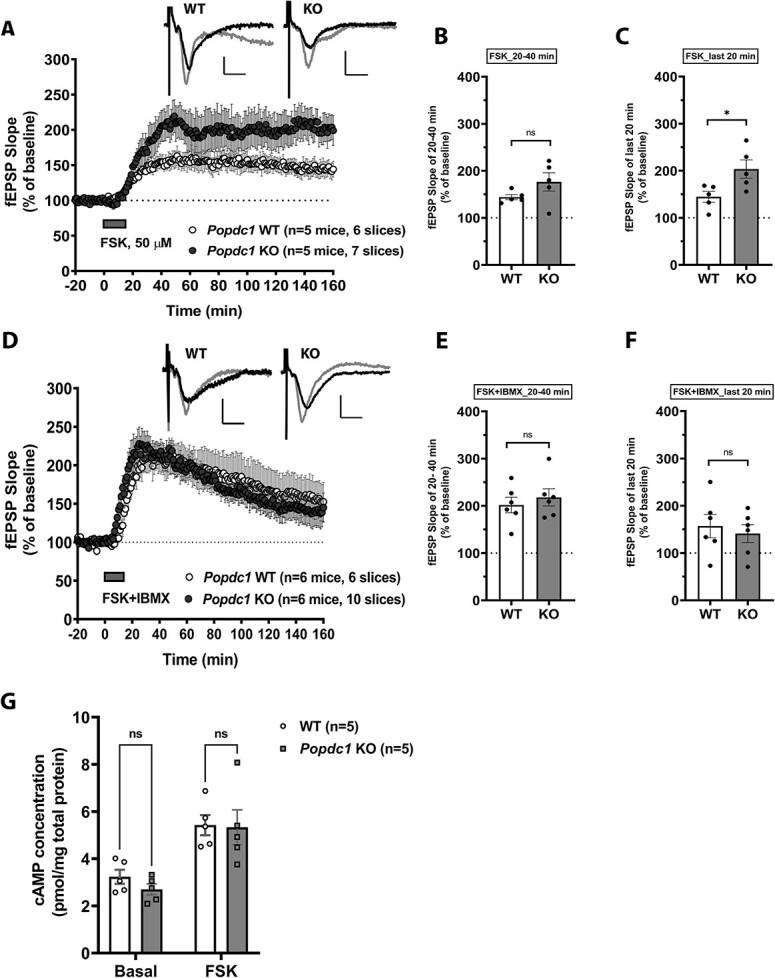
*Popdc1* KO mice show enhancement in potentiation induced by FSK but not with simultaneous inhibition of phosphodiesterases. (*A*) Persistent potentiation induced by bath application of 50 μM FSK is enhanced in slices from *Popdc1* KO mice (*n* = 5 mice; 3 males, 2 females) compared with WT littermates (*n* = 5 mice; 2 males, 3 females). (*B*) The mean fEPSP slope over the initial 20–40 min recordings from the drug application shows a trend for enhancement in *Popdc1* KO compared with WT; unpaired *t*-test, two-tailed, *t* = 1.595, df = 8, *P* = 0.149. (*C*) The mean fEPSP slope over the last 20 min recordings is significantly higher in *Popdc1* KO compared with WT; unpaired *t*-test, two-tailed, *t* = 2.611, df = 8, *P* = 0.0311. (*D*) Potentiation induced by coapplication of 50 μM FSK and 30 μM IBMX (FSK + IBMX) in slices from *Popdc1* KO mice (*n* = 6 mice; 5 males, 1 female) is indistinguishable from WT mice (*n* = 6 mice; 5 males, 1 female). (*E*) The mean fEPSP slope over the initial 20–40 min recordings from the drug application is similar in *Popdc1* KO and WT mice; unpaired *t*-test, two-tailed, *t* = 0.658, df = 10, *P* = 0.526. (*F*) The mean fEPSP slope over the last 20 min recordings shows no difference between *Popdc1* KO and WT mice; unpaired *t*-test, two-tailed, *t* = 0.5028, df = 10, *P* = 0.626. In *A* and *C*, representative fEPSP traces during baseline (black traces) and at the end of the recording period (gray traces) for each group are shown. Calibration bars for all traces: 2 mV vertical; 5 ms horizontal. (*E*) The total levels of cAMP measured in hippocampal slices of WT and *Popdc1* KO mice treated with 50 μM FSK or DMSO vehicle (Basal). Similar basal cAMP levels in *Popdc1* KO slices (*n* = 5 mice; 1 male, 4 females) compared with WT (*n* = 5 mice; 2 males, 3 females). Comparable cAMP levels in *Popdc1* KO and WT slices after FSK-stimulation; two-way ANOVA, *F*(1,16) = 0.456, *P* = 0.509; Šídák’s multiple comparisons tests between genotypes at basal or after FSK, *P* > 0.05. Errors bars represent SEM.

Because we observed a PKA-dependent enhancement in 1-train LTP in the *Popdc1* K*O* mice, we next examined the long-lasting potentiation induced by raising cAMP levels using FSK, a direct pharmacological activator of adenylyl cyclase (Chavez-Noriega and Stevens 1992; Huang and Kandel 1994; Otmakhov et al. 2004). Bath application of FSK (50 μM) resulted in significantly enhanced potentiation in slices from *Popdc1* KO animals compared with WT (Fig. 4*A*). The slow-onset potentiation induced by FSK showed a trend for enhancement in the KO slices shortly after the application (Fig. 4*B*; 20–40 min from the start; WT: 144 ± 5.5%, KO: 176.4 ± 19.5%; *t*-test, *P* = 0.149) and maintained at significantly higher levels than the WT slices (Fig. 4*C*; last 20 min; WT: 144.6 ± 11.5%, KO: 203.6 ± 19.5%; *t*-test, *P* = 0.031). Additionally, we tested the potentiation induced by FSK (50 μM) when applied along with a broad spectrum-phosphodiesterase inhibitor IBMX (30 μM) (Chavez-Noriega and Stevens 1992). At the concentration used, IBMX acts predominantly as a PDE inhibitor (Smellie et al. 1979) and results in further enhancement of FSK potentiation by augmenting cAMP levels (Vecsey et al. 2009; Li et al. 2018). The potentiation induced by FSK–IBMX was indistinguishable when compared between the slices from WT and *Popdc1* KO mice (Fig. 4*D*) and there was no difference either during early (Fig. 4*E*; *t*-test, *P* = 0.526) or late periods (Fig. 4*F*; *t*-test, *P* = 0.626). However, when we compared the potentiation induced by FSK and FSK–IBMX within each genotype, there was an intriguing finding. In the slices from WT mice, consistent with the earlier studies (Vecsey et al*.* 2009; Li et al. 2018), FSK–IBMX resulted in a robust early enhancement of potentiation compared with FSK alone (see Supplementary Fig. S4*A*). The potentiation shortly after the drug application (20–40 min after the start) was significantly higher (see Supplementary Fig. S4*B*; *t*-test, *P* = 0.014) with FSK–IBMX (202.9 ± 16.5%) than FSK (144.8 ± 5.7%) but the potentiation maintained at similar levels at the end (see Supplementary Fig. S4*C*; FSK 144.6 ± 11.5%, FSK–IBMX 157 ± 24.7%; *t*-test, *P* = 0.682). Intriguingly, in the slices from *Popdc1* KO mice (see Supplementary Fig. S4*D*), coapplication of IBMX, while not further enhancing the potentiation induced by FSK alone during earlier periods (see Supplementary Fig. S4*E*; FSK 177.8 ± 20.1%; FSK–IBMX 218.4 ± 17.9%; *t*-test, *P* = 0.165), rather resulted in a significant reduction in the potentiation towards the end of the recording (see Supplementary Fig. S4*F*; FSK 203.6 ± 19.5%, FSK–IBMX 141.3 ± 19.1%; *t*-test, *P* = 0.049). These results indicate that in *Popdc1* KO mice, the optimal cAMP levels permissive for enhancement of plasticity are achieved with FSK alone and further augmentation of cAMP with broader PDE inhibition seems to activate signaling pathways that are detrimental to the persistence of plasticity.

Because POPDC1 is a cAMP effector protein and we observed enhanced FSK-induced potentiation in the *Popdc1* KO slices, we tested if the loss of POPDC1 leads to enhanced cAMP levels. We used a cAMP ELISA assay to measure gross intracellular cAMP levels in the hippocampal slices from WT and *Popdc1* KO mice shortly after stimulation with FSK (50 μM) or vehicle (DMSO). The results showed no significant difference in the cAMP levels between the slices from WT and *Popdc1* KO mice (Fig. 4*G*; two-way ANOVA, *P* > 0.05), either at basal (vehicle DMSO-treated) conditions (WT: 3.238 ± 0.3 pmol/mg of total protein; KO: 2.702 ± 0.23 pmol/mg of total protein) or following FSK stimulation (WT: 5.426 ± 0.43 pmol/mg of total protein; KO: 5.331 ± 0.74 pmol/mg of total protein).

Taken together, these results indicate that POPDC1 plays a role in regulating local cAMP signaling in response to synaptic activity. Because changes in cAMP levels might be predominantly happening in localized domains near the synapses, estimation of gross cAMP levels may not have reliably captured these changes. Alternatively, the elevation in cAMP levels occurs either before or after the time-point used for sample collection in our experiment.

### Low-Frequency Stimulation-Induced LTP is Enhanced but LTD is not Altered in Popdc1 Knockout Mice

We further tested synaptic plasticity induced by theta-burst stimulation (TBS), a paradigm that has been proposed to resemble the rhythmic hippocampal electrical activity observed during spatial exploration (Buzsaki 2005). Because we observed an enhancement in 1-train LTP in the *Popdc1* KO mice, we used a weaker version of TBS containing only two bursts delivered at 5 Hz with each burst containing four 100 Hz pulses. This weak two-burst TBS protocol has been shown to induce a transient potentiation, whereas protocols containing more than five bursts result in persistent LTP (Kramar et al. 2009). Consistent with this, weak-TBS stimulation elicited a transient potentiation in slices from WT animals.

**Figure 5:**
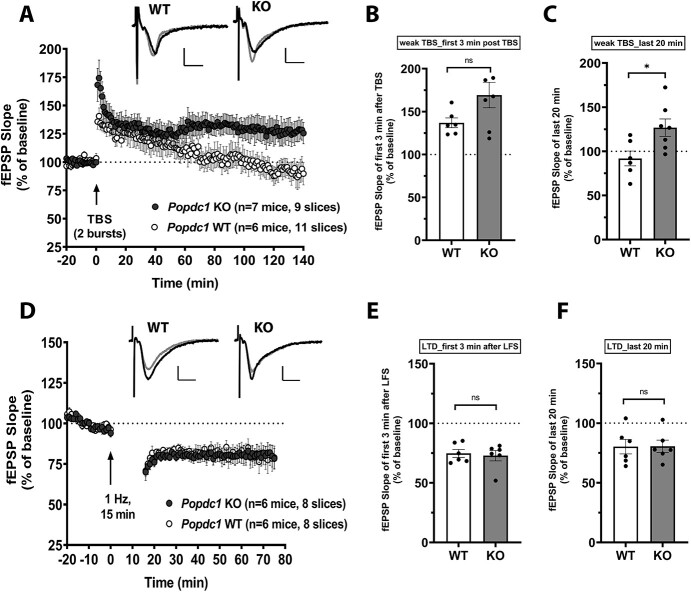
*Popdc1* KO mice show enhanced theta-burst LTP but show no alteration in LTD induced by LFS. (*A*) LTP induced by a weak TBS is enhanced in slices from *Popdc1* KO mice (*n* = 7 mice; 5 males, 2 females) compared with WT littermates (*n* = 6 mice; 5 males, 1 female). (*B*) Induction of LTP assessed by the mean fEPSP slope over the first 3 min after TBS shows a trend for enhancement in *Popdc1* KO compared with WT littermates; unpaired *t*-test, two-tailed, *t* = 1.909, df = 11, *P* = 0.083. (*C*) The mean fEPSP slope over the last 20 min recordings is significantly higher in *Popdc1* KO compared with WT littermates; unpaired *t*-test, two-tailed, *t* = 2.648, df = 11, *P* = 0.0226. (*D*) LTD induced by prolonged LFS (1 Hz, 15 min) is similar in slices from *Popdc1* KO mice (*n* = 6 mice; 3 males, 3 females) and WT littermates (*n* = 6 mice; 5 males, 1 female). (*E*) Induction of LTD assessed by the mean fEPSP slope over the first 3 min after LFS shows no difference between *Popdc1* KO and WT littermates; unpaired *t*-test, two-tailed, *t* = 0.354, df = 10, *P* = 0.731. (*F*) The mean fEPSP slope over the last 20 min recordings is similar in *Popdc1* KO and WT littermates; unpaired *t*-test, two-tailed, *t* = 0.0329, df = 10, *P* = 0.9743. In *A* and *D*, representative fEPSP traces at baseline (black traces) and at the end of the recording period (gray traces) for each group are shown. Calibration bars for all traces: 2 mV vertical; 5 ms horizontal. Errors bars represent SEM.

In slices from *Popdc1* KO mice, the weak-TBS stimulation resulted in enhanced LTP compared with slices from WT (Fig. 5*A*). The potentiation immediately after induction (first 3 min after TBS) was higher in the KO (169.3 ± 14.8%) compared with the WT (136.9 ± 5.7%) but the difference was not statistically significant (*P* = 0.083; two-tailed unpaired *t*-test; Fig. 5*B*). The persistence of LTP assessed by comparing the mean potentiation over the last 20 min recordings showed a significant enhancement in the *Popdc1* KO compared with the WT (Fig. 5*C*; KO: 126.9 ± 9.9%, WT: 91.9 ± 8.4%; two-tailed unpaired *t*-test, *P* = 0.0226). On the other hand, LTD induced with a prolonged low-frequency stimulation (LFS) protocol (Dudek and Bear 1992) showed no alterations between WT and *Popdc1* KO mice (Fig. 5*D*). The depression was not significantly different either immediately after induction (Fig. 5*E*; WT: 74.8 ± 3.3%, KO: 72.9 ± 4.4%; two-tailed unpaired *t*-test, *P* = 0.731) or at the end of the recording period (Fig. 5*F*; WT: 80.3 ± 6.1%, KO: 80.5 ± 5.2%; two-tailed unpaired *t*-test, *P* = 0.974). Although we used 2–3 months-old mice for the LTD experiments, under our stimulation parameters and experimental conditions involving long incubation periods and aCSF composition with Ca^2+^/Mg^2+^ ratio > 1, we could reliably induce LTD in slices from these mice using the 1 Hz, 15 min (900 pulses) protocol, as noted in previous studies (Dudek and Bear 1992; Heynen et al. 1996; Staubli and Ji 1996; Billard 2010; Delgado et al. 2018).

**Figure 6:**
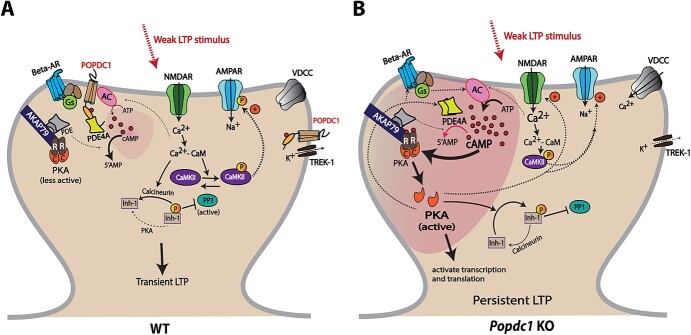
A schematic depiction of a working model to explain the enhanced LTP observed in *Popdc1* KO mice in response to submaximal high-frequency stimulation. (*A*) In hippocampal neurons of WT mice, POPDC1 protein localizes near the synapse in proximity to other synaptic proteins involved in cAMP signaling including beta-adrenergic receptors (beta-AR), adenylyl cyclases (AC), phosphodiesterases such as PDE4A, and the two-pore potassium channel TREK-1. POPDC1 interacts with the PDE4A long isoforms through their UCR1 region, thus sequestering them in monomeric form that is highly active due to its “open” conformation. A weak synaptic stimulus that induces a transient LTP leads to influx of Ca^2+^ through NMDAR and activation of Ca^2+^-calmodulin (CaM)-dependent kinase II (CaMKII). Active CaMKII phosphorylates AMPA receptors (AMPAR) increasing the channel conductance, and also leads to increased synaptic trafficking of AMPARs. The stimulation might also induce modest increase in cAMP levels through Ca^2+^-CaM-dependent AC activation, but the cAMP is quickly degraded by the highly active PDE4 monomers, thereby preventing activation of AKAP79-anchored PKA and other cAMP effectors. Ca^2+^-CaM-dependent activation of calcineurin keeps inhibitor-1 (Inh-1) dephosphorylated, in turn resulting in active PP1. Low local cAMP levels and active phosphatase cascades only allow a short-lasting LTP. (*B*) In the hippocampal neurons of *Popdc1* KO mice, possible alterations in presynaptic mechanisms and/or enhanced NMDAR activity lead to efficient AC activation and cAMP elevation in response to LTP stimulus. The absence of POPDC1 may lead to increased formation of PDE4A dimers that are less efficient at cAMP degradation due to the “closed” conformation and are spatially less restricted near the synapses. This results in an accumulation and persistence of local cAMP produced in response to weak LTP-inducing stimulus. The increase in cAMP levels leads to activation of locally anchored PKA, which phosphorylates many of its targets such as AMPAR, NMDAR, beta-AR etc., and activates transcription and translation. This, along with inhibition of the phosphatase cascade and possibly other mechanisms involving AC inactivation, TREK-1 and voltage-dependent calcium channels, results in persistent LTP.

## Discussion

The cAMP-mediated signaling pathways play a crucial role in the persistent forms of synaptic plasticity (Abel et al. 1997; Abel and Nguyen 2008). Given the central role of cAMP signaling in many cellular processes, the activation of downstream targets is spatiotemporally regulated. This is achieved in a number of ways: via controlling the generation of cAMP by adenylyl cyclases, by regulating cAMP degradation through PDEs and by the subcellular targeting of cAMP effector proteins with the help of a number AKAP proteins (Zaccolo et al. 2021). The cAMP effector proteins PKA, EPAC, and hyperpolarization-activated cyclic nucleotide-gated cation channel have been well characterized in neurons (Kelly 2018). The POPDC proteins are a recent addition to this group of cAMP effectors (Brand 2018) and until now their function in the nervous system has not been studied. In this study, we investigated the role of POPDC1 in synaptic plasticity in the hippocampal CA3-CA1 Schaffer collateral synapses using mice lacking *Popdc1* (Andrée et al. 2002). We show that *Popdc1* is expressed widely in different brain regions including all the subregions of hippocampus. Moreover, our initial findings suggest that POPDC1 protein seems to be enriched in crude hippocampal synaptosomes, thereby potentially supporting its function in synaptic plasticity. Nevertheless, additional approaches to confirm the cellular and subcellular localization of POPDC1 in neurons are needed. Future studies investigating the function of POPDC1 in other brain regions might also uncover additional roles for this protein.

Our results show that *Popdc1* KO mice exhibit enhanced LTP induced by a single HFS train. Similar enhancement has also been reported following pharmacological enhancement of hippocampal cAMP levels (Barad et al. 1998), in mice expressing an inhibitor of ATF4 (CREB-2) and C/EBP proteins (Chen et al*.* 2003) and following genetic inhibition of the phosphatase calcineurin (Malleret et al*.* 2001). LTP enhancement was not apparent in response to strong tetanization protocols, possibly due to the saturating magnitude of potentiation induced by such paradigms or, in other words, due to a “ceiling effect.” Persistent LTP observed in the *Popdc1* KO following a single HFS train was found to be dependent on PKA activation. This is in line with previous studies implicating a crucial role for PKA in persistent forms of LTP, where PKA activation functions like a gate between transient and persistent forms of LTP (Blitzer et al. 1995; Abel et al. 1997). We also observed similar LTP enhancement in hippocampal slices of *Popdc1* KO mice subjected to a subthreshold TBS protocol. Persistent LTP induced by stronger TBS protocols (those involving more than five bursts) has been shown to be dependent on PKA (Nguyen and Kandel 1997) and PKA anchoring by AKAPs (Nie et al. 2007). Additionally, enhanced FSK-induced potentiation in the *Popdc1* KOs further indicates an augmented activation of the cAMP–PKA signaling in the absence of POPDC1. A trend for enhanced potentiation immediately following LTP induction in response to all these stimulation paradigms suggests that loss of *Popdc1* results in a lowered LTP induction threshold. This could involve alterations in N-methyl-D-aspartate (NMDA) receptor (NMDAR) function (Otmakhova et al. 2000; Skeberdis et al. 2006), altered AMPA receptor trafficking or presynaptic mechanisms (Trudeau et al. 1996; Park, Havekes, et al. 2014; Park, Volianskis, et al. 2014). In future studies, it will be important to confirm the involvement of PKA activation in the enhanced LTP using, for example, additional inhibitors such as H89 or PKI peptide and to test if the LTP enhancement is dependent on NMDAR and involves altered gene expression and protein synthesis.

Although our fEPSP input–output response and PPF data indicate no gross changes in the basal synaptic transmission in *Popdc1* KO mice, dampened fEPSP suppression dynamics during high-frequency stimulation seems to suggest alterations in afferent excitability, presynaptic release, or Ca^2+^ influx through NMDARs (Grover et al. 2009; Kim et al. 2012; Regehr 2012; Helassa et al. 2018). Alternatively, changes in neuronal or dendritic excitability could underlie the altered response during tetanus. Our work in cardiac myocytes has identified the two-pore potassium channel TREK-1 as an important effector of cAMP-dependent POPDC1 function in the heart (Froese et al*.* 2012; Schindler et al*.* 2016). In neurons, TREK-1 plays a crucial role in the maintenance of the resting membrane potential and thereby neuronal excitability (Honore 2007; Enyedi and Czirjak 2010). Studies have also implicated TREK-1 in synaptic plasticity and memory (Weng et al. 2016; Cai et al. 2017; Wang et al. 2020). Therefore, it is possible that POPDC1 modulation of potassium channels, either directly or via cAMP–PKA pathway, could have a role in the dampened fEPSP suppression dynamics in *Popdc1* KO mice. In future studies, it will be informative to directly test the role of POPDC1 in modulation of NMDAR function, EPSP-Spike (E-S) potentiation and neuronal excitability characteristics.

The finding that loss of POPDC1 results in enhancement of LTP in response to several stimulation paradigms suggests that POPDC1 might function as a molecular brake to constrain plasticity in the hippocampal CA1 region. Given its demonstrated role as a cAMP-effector protein (Schindler et al. 2016; Swan et al. 2019), evidence for its direct interaction with cAMP-specific phosphodiesterases (Tibbo et al. 2020) and with the brain-enriched regulatory subunit isoforms (PR61α/B56) of the phosphatase PP2A (Parang et al. 2017), POPDC1 seems to function in regulating cAMP–PKA–PDE signaling dynamics in local domains, in response to synaptic activity. The fact that the input-specificity of LTP is still preserved in the absence of POPDC1 further supports that local, activity-dependent mechanisms underlie the enhancement and that regulatory feedback mechanisms are not widely altered. The intriguing late decay observed in the potentiation induced by FSK + IBMX in *Popdc1* KO mice could be due to overt augmentation of cAMP signaling leading to recruitment of other pathways that disrupt LTP maintenance. Our results showing no alteration of LFS-induced LTD in *Popdc1* KO mice indicate that POPDC1 is preferentially involved in the regulation of LTP and not LTD. Nevertheless, other forms of LTD induced by other electrical or chemical stimulation paradigms need to be investigated to be conclusive about this preferential involvement.

We did not find significant differences in gross cAMP levels in the hippocampal slices of *Popdc1* KO mice either at basal activity or following FSK stimulation, but we suspect that it could be due to limitations of the ELISA assay in capturing local changes in cAMP nanodomains or due to the timing of sample collection. It will be interesting to investigate the role of POPDC1 in local cAMP dynamics during synaptic activity using imaging approaches based on genetically encoded cAMP sensors (Schleicher and Zaccolo 2018). Since POPDC1 displays high and selective cAMP-binding affinity similar to that of PKA (Froese et al. 2012; Brand 2018), it is possible that it might contribute to the spatial control of cAMP diffusion by binding to cAMP and limiting the activation of other cAMP effector proteins. Alternatively, POPDC1 might form complexes with other proteins to regulate their post-translational modifications in a cAMP-dependent manner, either directly or indirectly though the modulation of PDE or phosphatase activity. Currently, we cannot definitively distinguish between different models of POPDC1 action (Swan et al. 2019) in relation to hippocampal synaptic plasticity and determining the POPDC1 interactome in neurons would greatly facilitate this.

In the light of our current results, evidence from relevant interactions of POPDC1 and based on prominent mechanisms known to mediate hippocampal synaptic plasticity, we propose a working model for the role of POPDC1 in synaptic plasticity. In WT mice (Fig. 6*A*), POPDC1 localizes near the synapses and interacts with PDE4A long isoforms through their UCR1 region (Tibbo et al*.* 2020), thus acting like a PDE4A anchor to result in a spatially restricted cAMP signal. This interaction might prevent PDE4A dimerization, either directly by affecting the conformation or indirectly by sequestering the monomers, because the UCR1 region is crucial for the dimerization (Bolger et al. 2015). The monomeric PDE4s have an “open” conformation and show much greater activity towards cAMP degradation compared with PDE4 dimers with a preferential “closed” conformation (Perry et al. 2002; Cedervall et al. 2015). Additionally, beta-arrestins show higher affinity for open monomers of PDE4 than the closed dimers for recruiting them to the ligand-occupied beta2-adrenergic receptor, resulting in downregulation of cAMP signaling (Perry et al. 2002; Baillie et al. 2003). Overall, POPDC1 and the anchored PDE4A monomers near the synapse might be acting to limit elevation of local cAMP levels in response to weaker LTP-inducing stimuli, thereby preventing the activation of other cAMP effectors, such as PKA. Further, in the absence of PKA activation, inhibitor-1 is kept in a dephosphorylated state by the action of calcineurin, which leads to the activation of protein phosphatase 1 (PP1). These phosphatase cascades would make sure that the LTP stimulus-induced synaptic changes, such as phosphoCaMKII-mediated AMPAR phosphorylation, are transient. In *Popdc1* KO mice (Fig. 6*B*), the absence of POPDC1 may lead to increased PDE4A dimerization and loss of spatially restricted localization of PDE4A near the synapse. Now, in response to the LTP-inducing stimulus, cAMP can diffuse more widely, and the signal may last longer because PDE4 dimers have reduced catalytic activity and may not be properly localized. These changes may cause enhanced cAMP signaling leading to increased activation of cAMP effectors, such as PKA. Active PKA may then phosphorylate many of its plasticity-related targets, including NMDARs, AMPARs, Inhibitor-1, leading to the activation of transcription and translation and ultimately persistent LTP. Furthermore, loss of POPDC1 might also alter the activity of certain potassium channels, presynaptic mechanisms and thereby neuronal excitability.

Taken together, our findings identify POPDC1, a unique cAMP effector protein, as a novel player involved in the regulation of hippocampal synaptic plasticity and pave the way for future studies aimed at understanding its function in learning and memory. Although it is indeed tantalizing to investigate the role of POPDC1 in memory, some of the phenotypes displayed by the constitutive *Popdc1* KO mice, such as the stress-induced sinus node bradycardia (Froese et al. 2012) and an overall hyperexcited state, makes this mouse line not suitable for use in behavioral memory tasks. The interaction of POPDC1 with PDE4A5 (Tibbo et al. 2020) and its potential involvement in the regulation of hippocampal synaptic plasticity is an exciting avenue given that phosphodiesterase inhibition is a promising strategy to enhance synaptic plasticity and memory (Barad et al. 1998; Baillie et al. 2019). Further, previous studies have implicated PDE4A5 in mediating the impact of sleep deprivation on hippocampal synaptic plasticity and memory (Vecsey et al. 2009; Wong et al. 2019) and it will be exciting to investigate if POPDC1 has any role in sleep-related synaptic plasticity. Finally, given that POPDC1 seems to have a role in regulating LTP induction threshold while aging is associated with increased LTP threshold and impairments in persistence of LTP (Bach et al. 1999), it will be interesting to examine whether blocking POPDC1 function might be able to rescue age-related LTP deficits. We are in the process of generating conditional KO of *Popdc1* and these would be invaluable in investigating the function of POPDC1 protein in specific brain regions involved in learning and memory.

## Author Contributions

M.S.S. and L.R. performed the electrophysiology experiments. M.S.S. performed the cAMP assays, analyzed the data, and wrote the manuscript with inputs from other authors. T.A. supervised electrophysiology experimental design, data analysis, and provided inputs to the manuscript. R.F.R.S. performed the Western blot analysis and L.F. performed the *LacZ* immunohistochemistry. L.R, K.P.G., and K.M performed some of the initial analysis of hippocampal function and memory in *Popdc1* KO mutants. T.B. initiated this study and was involved in data interpretation and manuscript preparation.

## Funding

National Institutes of Health (R01 MH 117964 to T.A.); Medical Research Council (MR/J010383/1) and British Heart Foundation (PG14/46/3091 and PG19/13/34247) to T.B. T.A. is supported by the Roy J. Carver Chair in Neuroscience. L.R. is supported by the Belgian Queen Elisabeth Medical Foundation.

## Notes

We thank Ursula Herbort-Brand for the excellent technical assistance. We also thank Tania Chatterjee Chowdhury, Jia Ern Ong and Achala Thippeswamy for their help with colony maintenance and genotyping. *Conflicts of Interest*: The authors declare that there are no conflicts of interest.

## Supplementary Material

Shetty_et_al_CerCor-2021-00347_R1_Final_Supplementary_Data_bhab426
