# Supplementary material for: Mice lacking the cAMP effector protein POPDC1 show enhanced hippocampal synaptic plasticity
Source: Cereb Cortex. 2021 Dec 23;32(16):3457–71. doi: 10.1093/cercor/bhab426 (PMC9376866; doi:10.1093/cercor/bhab426)
Supplement: Shetty_et_al_CerCor-2021-00347_R1_Final_Supplementary_Data_bhab426 [file shetty_et_al_cercor-2021-00347_r1_final_supplementary_data_bhab426.pdf]

Supplementary Data:

CerCor-2021-00347

Mice lacking the cAMP effector protein POPDC1 show enhanced hippocampal synaptic plasticity

Mahesh Shivarama Shetty, Laurence Ris, Roland F. R. Schindler, Keiko Mizuno, Laura Fedele, Karl Peter Giese, Thomas Brand and Ted Abel

Supplementary Figure S1.

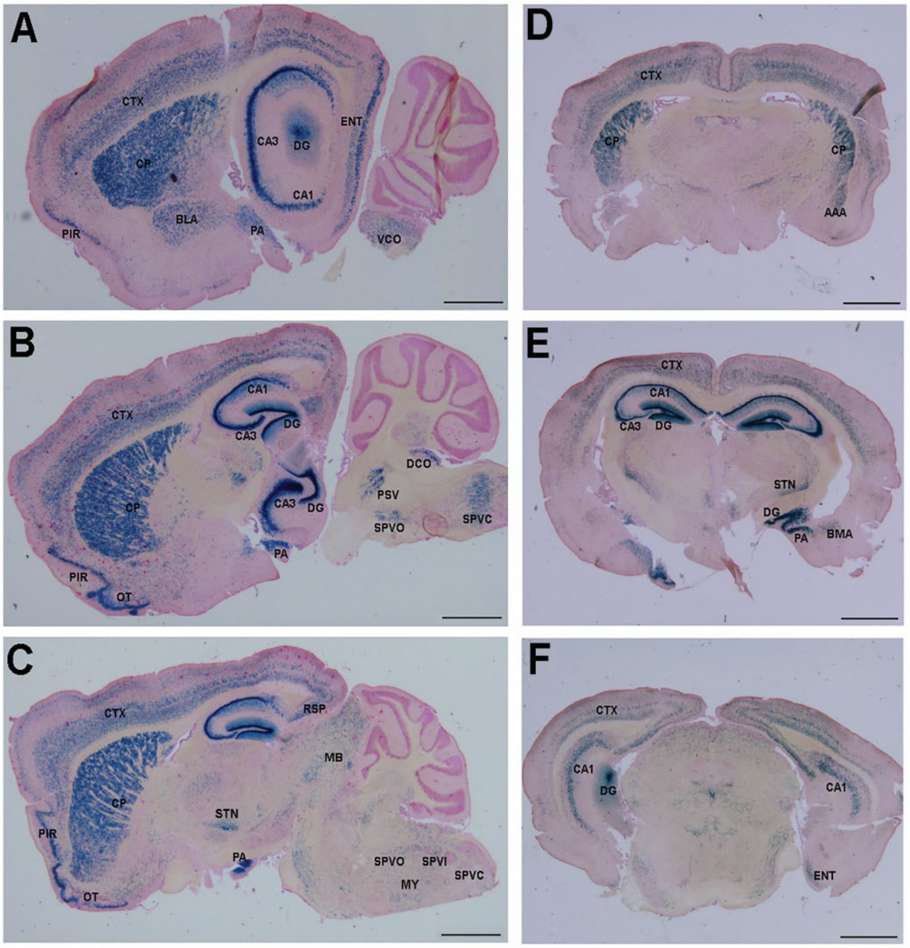

Supplementary Figure S1. Expression of *Popdc1* in the mouse brain. X-gal staining of sagittal sections **(A-C)** and coronal sections **(D-F)** of 3-months old heterozygous *Popdc1-LacZ* mice showing expression in various regions of the brain. Abbreviations: AAA- antero amygdalar area, BLA – basolateral amygdala , BMA – basomedial amygdalar nucleus, CA1/CA3 - cornu ammonis 1/-3, CP – caudoputamen, CTX-cerebral cortex, DCO – dorsal cochlear nucleus, DG – dentate gyrus, ENT – entorhinal area, MB – midbrain, MY – medulla, OT - olfactory tubercle, PA – posterior amygdala, PIR – piriform area, PSV - principal sensory nucleus of the trigeminal, SPVO/SPVC/SPVI – spinal nucleus of the trigeminal oral part/-caudal part/-interpolar part, STN - subthalamic nucleus, VCO – ventral cochlear nucleus. Scale bar: 1 mm.

Supplementary Figure S2.

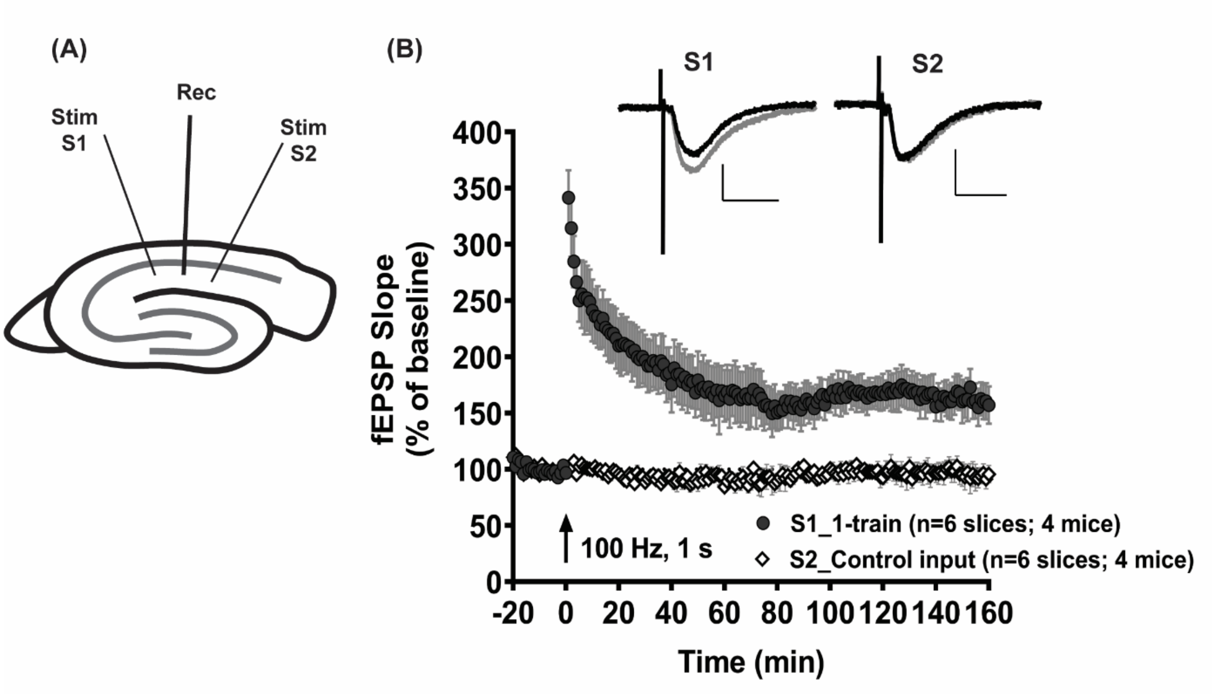

Supplementary Figure S2. Enhanced 1-train LTP in *Popdc1* KO mice is input-specific. (A) Schematic representation of a hippocampal slice showing the location of electrodes in the CA1 stratum radiatum. Two electrodes S1 and S2 are used to stimulate two independent Schaffer collateral inputs onto a population of neurons and the fEPSP responses are recorded with a common recording (Rec) electrode. **(B)** In the slices from *Popdc1* KO mice, LTP induced by a single 100 Hz train (1-train LTP) is persistently enhanced in an input-specific manner. Only the input S1 that received the 1-train stimulation shows LTP while the test responses in control input S2 remain stable for the entire recording period. Representative fEPSP traces are shown in the insets for each input at baseline (black trace) and at the end of the recording (grey trace). Calibration bars for traces: 2 mV vertical, 5 ms horizontal. The error bars represent SEM.

Supplementary Figure S3.

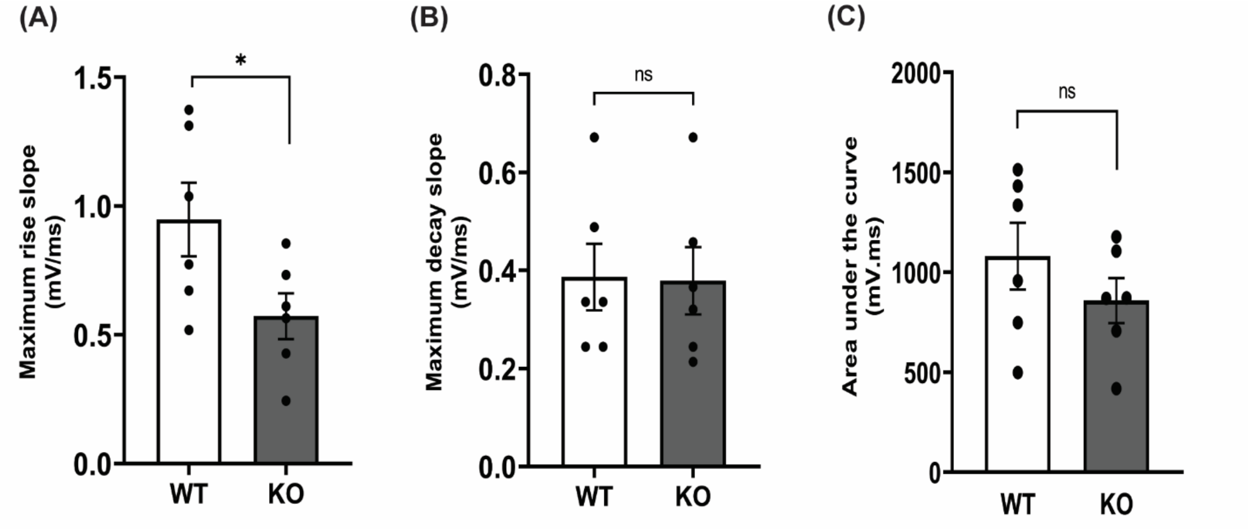

Supplementary Figure S3. Altered fEPSP response during 1-train stimulation in *Popdc1* KO mice. Characteristics of the cumulative fEPSP response during 1-train stimulation (100 Hz, 1 s, 100 stimuli). Data from multiple slices of the same mouse are averaged. For all the graphs, WT: n=6 mice, 8 slices (2 males, 4 females); KO: n=6 mice, 7 slices (4 males, 2 females). **(A)** The mean maximum rise slope of the response was significantly smaller in the *Popdc1* KO (0.572±0.09 mV/ms) compared to the WT (0.948±0.14 mV/ms) (unpaired t-test, t=2.233, df=10, P=0.049). **(B)** The mean maximum decay slope of the response was similar in *Popdc1* KO (0.387±0.07 mV/ms) and WT (0.379±0.07 mV/ms) (unpaired t-test, t=0.079, df=10, P=0.938). **(C)** The mean area under the curve of the response was slightly smaller in *Popdc1* KO (1081±167 mV.ms) and WT (858.5±112 mV.ms) (unpaired t-test, t=1.105, df=10, P=0.295). In all the figures, data are presented as mean ± SEM.

Supplementary Figure S4.

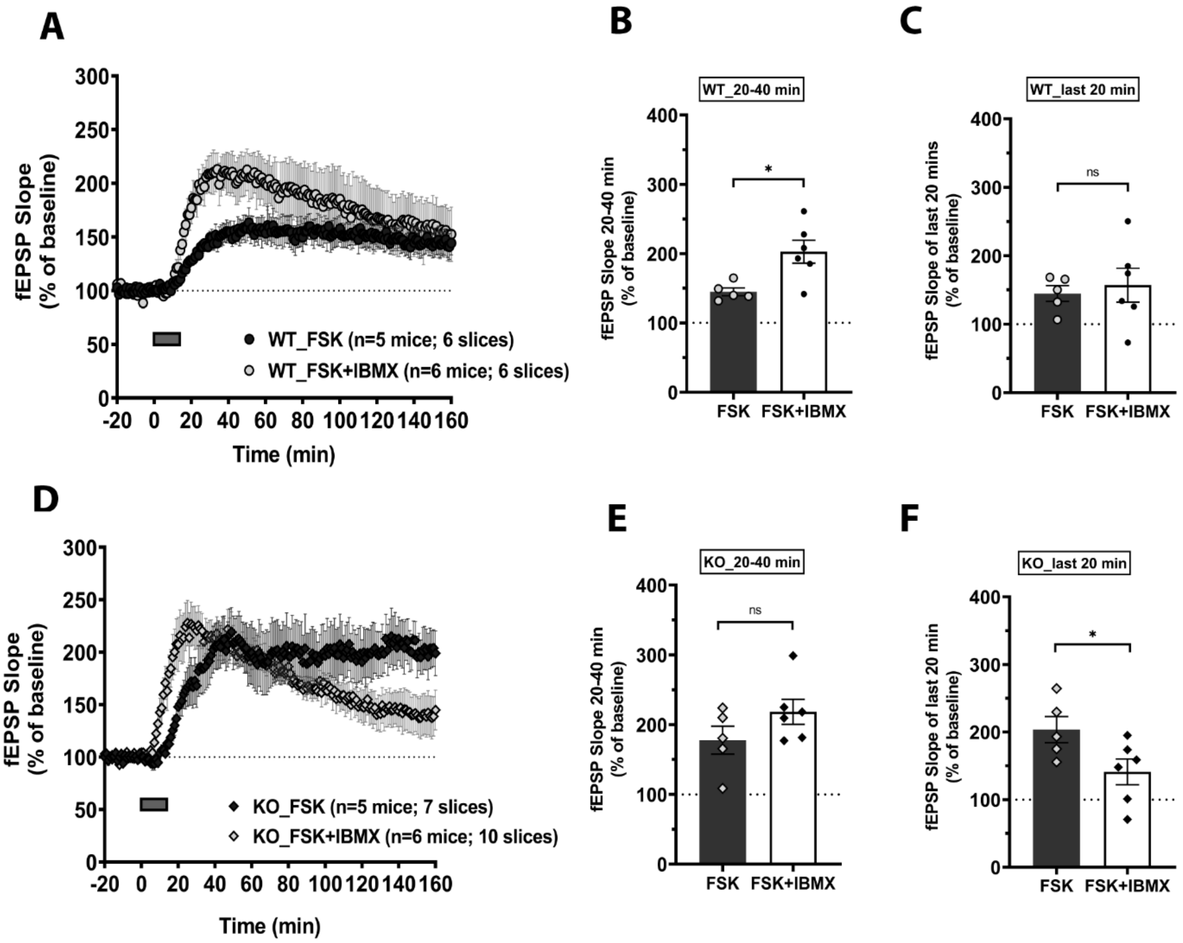

Supplementary Figure S4. Negative impact of broad phosphodiesterase inhibition on enhanced forskolin potentiation in the *Popdc1* KO mice. (A-C) In the slices from WT mice, bath application of the adenylyl cyclase activator forskolin (FSK, 50 μM) results in slow-onset persistent potentiation. Co-application of FSK with a broad-spectrum phosphodiesterase (PDE) inhibitor IBMX (30 μM) leads to an early enhancement in FSK potentiation. Potentiation during 20-40 min with FSK+IBMX (202.9±16.5%) is significantly higher than FSK alone (144.8±5.7%) (unpaired t test, t=3.059, df=9, P=0.014). Potentiation during the last 20 min is similar with FSK+IBMX (157±24.7%) and FSK (144.6±11.5%) (unpaired t test, t=0.423, df=9, P=0.682). **(D-F)** In the slices from *Popdc1* KO mice, FSK alone induces persistently enhanced potentiation compared to WT and FSK+IBMX leads to a late decay in the potentiation. While potentiation during 20-40 min is similar with FSK+IBMX (218.4±17.9%) and FSK alone (177.8±20.1%) (unpaired t test, t=1.51, df=9, P=0.165), during the last 20 min there is a significant decay of FSK+IBMX potentiation (141.3±19.1%) and FSK (203.6±19.5%) (unpaired t test, t=2.271, df=9, P=0.049). In all the figures, error bars represent SEM.
